# Human Epididymis Protein 4 Levels in Neonates with Respiratory Disorder

**DOI:** 10.1155/2020/1509379

**Published:** 2020-04-02

**Authors:** Piotr Surmiak, Martyna Szymkowiak, Małgorzata Baumert

**Affiliations:** Department of Neonatology, Medical University of Silesia in Katowice, Faculty of Medical Sciences, Katowice 40-055, Poland

## Abstract

**Results:**

There were no differences found in the HE4 levels determined for the mothers' blood samples and umbilical cord blood samples in all investigated groups. In comparison with healthy children, the elevated HE4 levels were observed in neonates with TTN. Significant positive correlation between HE4 and CRP as well as PCT levels was observed in all investigated neonates. The receiver operating characteristic (ROC) curve analysis demonstrated the cut-off value for the serum HE4 in the researched neonates at the level of 318.5 pmol/L, yielding the sensitivity of 73.9% and specificity of 66.7% for the early diagnosis of TTN.

**Conclusions:**

Serum HE4 could be considered as a candidate biomarker for the early diagnosis of pulmonary dysfunction in the newborns.

## 1. Introduction

Transient tachypnea of the newborn (TTN) is one of the most common causes of respiratory distress in neonatal period, affecting 0.5% to approximately 5% of all late preterm and term neonates in the immediate postdelivery period [1–3]. TTN called wet lungs is a common self-limited disease of term newborns that results from delayed lung fluid clearance and sometimes affects babies during the first hours of life [4, 5]. The diagnosis of TTN in early postnatal period remains problematic for clinicians. The most typical presenting symptoms, tachypnea and respiratory distress and the need for supplemental oxygen, are common among most neonatal respiratory disorders, and unfortunately, there exist no reliable diagnostic tests for TTN.

Even though there is broad interest in the biomarker research, the scientific investigation has yielded only a few new indicators (particularly cancer biomarkers) that seem valuable for the clinical use in recent years. The search for the new markers that could help to detect a specific disease is driven by the hope for the early disease detection and immediate treatment implementation to achieve the best possible results in the disease treatment. For that reason, the markers should be highly specific and sensitive.

The serum human epididymis protein 4 (HE4) is one of the biomarkers recently included in the pipeline of clinical testing [6]. HE4 (also known as the WAP four-disulfide core domain protein 2, WFDC2) is a N-glycosylated protein, which is encoded by the gene located on the chromosome 20q12-13.1. The protein was first identified as a transcript exclusively expressed in the epididymis and concerned with the maturation of sperm [7]. HE4 is a good example of a candidate biomarker originating from the gene expression analysis that was later translated into the clinical practice.

In recent years, the number of articles on the diagnostic utility of HE4 levels (e.g., in the ovarian cancer diagnostics) has considerably increased [8]. Some authors reported the elevated HE4 levels in the cases of other diseases (such as renal fibrosis and chronic kidney disease, cystic fibrosis, lung cancer, and the degree of the pulmonary dysfunction) [9–13].

Based on above, the authors of this study decided to investigate the HE4 levels in the umbilical cord blood and venous blood of the neonates with respiratory disorder (transient tachypnea of the newborn) and healthy newborns.

## 2. Material and Methods

### 2.1. Study Population

The researched sample came from a single-center prospective cohort study, approved by the Bioethics Committee of the Medical University of Silesia in Katowice (no. KNW/022/KB1/151/I/16/17). All the parents provided a signed informed consent before recruitment into the research. The neonatal care and monitoring were performed as part of the standard hospital protocol. Among the 1132 children born in the Department of Neonatology (Medical University of Silesia in Katowice, Poland) between May 1, 2017, and May 31, 2018, 51 full-term newborn babies (37-41 hbd) were enrolled in this study.

The investigated neonates were divided into two groups: 23 neonates with the respiratory insufficiency (*transient tachypnea of the newborn*, *TTN*) as the study group and 28 newborn babies of healthy mothers constituted the control group (CG). All neonates suffered TTN immediately after delivery, and the diagnosis was based on the clinical symptoms and chest X-ray.

The neonates that were small for the gestational age; the ones with congenital abnormalities or chromosomal anomalies, metabolic disorders, and congenital infections; and the ones from multiple pregnancies were excluded from the study. Newborns from mothers who suffered from diabetes mellitus and asthma were also excluded from this investigation. The information on the subject demographics, clinical history, and relevant comorbidities was collected upon the enrollment.

The demographic and perinatal characteristics of the study population are presented in Table 1.

### 2.2. Research Methods

During the delivery, 2 mL of the maternal venous blood and 1 mL of the umbilical artery blood were collected. The neonatal blood samples (0.5 mL) were collected from the peripheral vessels during a routine blood sampling procedure 24 hours after the birth.

The C-reactive protein (CRP) and procalcitonin (PCT) concentrations were determined for all the examined neonates immediately after the delivery. The C-reactive protein values were measured with the immunoturbidimetry method (Beckman Coulter AU analyzer). The procalcitonin levels were determined with the electrochemiluminescence method (ECLIA) (Cobas E 601 analyzer). The blood samples for the HE4 measurements were collected, centrifuged for 10 minutes (2500 rotations/min), and stored at -80°C until the full analysis was performed. The serum HE4 levels were determined with the sandwich-enzyme immunoassay (ELISA) (BioVendor kit; Brno, Czech Republic). The ELISA allows for quantitative measurements of the HE4 levels in the serum. It has a detection limit of 0.15 pmol/L.

### 2.3. Statistical Analysis

The normal distribution of the data was verified with the Shapiro-Wilk test. The continuous variables among the groups were compared with the Kruskal-Wallis test or the Mann-Whitney *U* test due to the nonparametric nature of the data. The categorical variables were compared with the chi-squared test. The quantitative variables are presented as a median with 95% confidence intervals for the median (95% CI), whereas the qualitative variables are given as percentage values. The Spearman correlation coefficients were calculated between the laboratory HE4 measurements and clinical variables. The HE4 levels are expressed as a median and 95% confidence intervals for the median (95% CI) due to the nonparametric nature of the data.

The predictive performance was assessed by means of the area under the receiver operating characteristic (ROC) curve. The ROC curves and area under the curves (AUC) with Youden index were used to assess the diagnostic accuracy of the serum HE4 for predicting transient tachypnea of the newborn.

The statistical analysis was based on the standard procedures available in STATISTICA 13.3 (Statsoft Polska Inc.) and MedCalc Software 12.7.4. The statistical inferences were based on the level of significance (*p* < 0.05).

## 3. Results

The mothers and newborns from the study group (TTN) were compared with the controls. The demographic and perinatal characteristics were taken into consideration. There were no significant differences in birth weight, gender, and Apgar score between the group of neonates with TTN compared with controls (Table 1).

We did not observe any differences in the HE4 concentrations in the mothers' blood samples (*p* = 0.65) or in the umbilical cord blood samples (*p* = 0.14) between the TTN and control groups. The significantly higher HE4 concentration values in the neonatal blood (collected 24 hours after the delivery) were observed in comparison with the level determined for the umbilical cord blood in both investigated groups (*p* < 0.0001).

**Figure 1:**
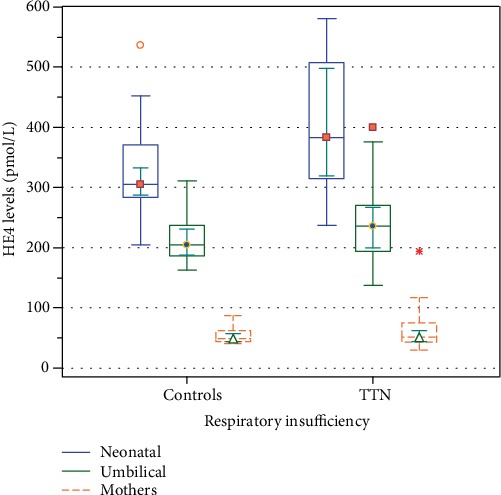
Serum HE4 levels in mothers' blood samples, umbilical cord blood samples, and neonatal blood samples collected 24 hours after the delivery in the neonates with respiratory insufficiency (transient tachypnea of the newborn, TTN) and controls. Results presented as a median as well as 95% confidence intervals for median and extreme values.

When compared with controls, 23 neonates with the respiratory insufficiency (TTN) demonstrated significantly higher HE4 levels (*p* = 0.003). The respective results are presented in Table 2 and Figure 1.

There were no differences among the investigated groups when it comes to the CRP and PCT levels in the mothers' and neonatal venous blood samples collected 24 hours after delivery in the TTN and control groups (Table 2).

When taking into account the neonates' gender, there were no differences in the HE4 concentrations in either the umbilical cord blood samples or the neonatal venous blood samples collected 24 hours after the delivery (Table 3).

**Figure 2:**
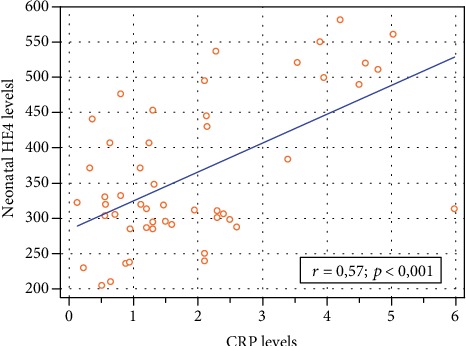
Correlation chart of the serum HE4 (pmol/L) and CRP (mg/L) levels in the neonates' blood collected 24 hours after the delivery.

**Figure 3:**
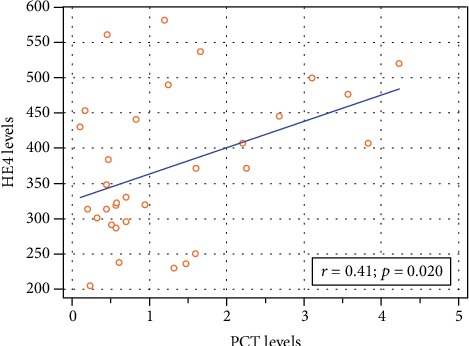
Correlation chart of the serum HE4 (pmol/L) and PCT (ng/mL) levels in the neonates' blood collected 24 hours after the delivery.

First, the HE4, CRP, and PCT levels were correlated in all the groups. A significant positive correlation was detected between the HE4 and CRP values (*r* = 0.57; *p* < 0.001) in all neonates. The results are presented in Figure 2. Additionally, significant correlation was found between the HE4 and procalcitonin concentrations (*r* = 0.41; *p* = 0.02) in the studied newborns, shown in Figure 3.

We did not observe any correlation between CRP, PCT, and HE4 levels in mothers' blood samples.

**Figure 4:**
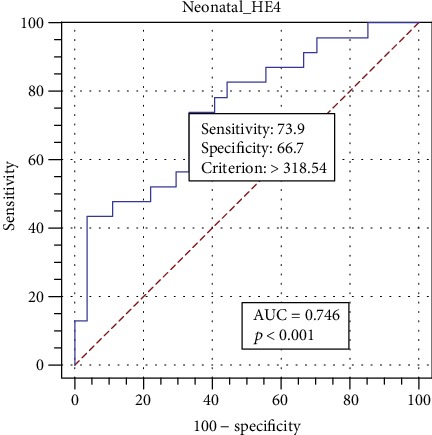
Receiver operating characteristic (ROC) curve used to determine the serum HE4 cut-off value with the Youden index for the TTN detection. The area under the curve (AUC) value for the serum HE4 was 0.75 (95% CI 0.60-0.86); *Z* statistic 3.50; +LR 2.22 (95% CI 1.20-4.00); -LR 0.39 (95% CI 0.20-0.79) and *p* < 0.001; Youden index *J* = 0.41.

To determine the performance of HE4 as a prognostic biomarker, the AUC analysis with Youden index was performed. The cut-off value with the Youden index for the serum HE4 in the neonates 24 hours after the delivery was 318.5 pmol/L, yielding the sensitivity of 73.9% (95% CI 51.6–89.8%) and specificity of 66.7% (95% CI 46.0–83.5%) for the early diagnosis of TTN (area under the ROC curve, AUC = 0.75 [95% CI 0.60–0.86]; *Z* statistic 3.5; *p* < 0.0001; Youden index *J* = 0.41). The results are given in Figure 4.

## 4. Discussion

Cancer biomarkers have become an important tool in the treatment of cancer patients, particularly in the monitoring of patients during and after the treatment [14, 15]. Recently, the serum HE4 has been identified as a diagnostic and prognostic biomarker for lung cancer, lung malignancies, ovarian carcinoma, and endometrial carcinomas [16, 17]. The HE4 levels may also be elevated in certain noncancer diseases, such as the chronic kidney disease or heart failure severity [9, 18]. Significant increases in the expression and localization of the WFDC2 were observed in patients with cystic fibrosis. Additionally, the abnormal HE4 concentrations were found in the cases of severe bronchitis, asthma, pneumonia, and inflammatory processes [10, 19]. Nonetheless, little is known about the fluctuation of the cancer marker concentrations in a healthy reference population, especially in children and neonates [20, 21].

As there are no reference intervals, the authors of this article decided to study the full-term neonates with respiratory disorder (TTN) so as to evaluate the HE4 concentration in the umbilical cord blood and venous blood (24 hours after the delivery). In the study, the mean HE4 values determined for the 51 examined newborn babies were 355.2 pmol/L with 95% confidence intervals for the median of 301.1 pmol/L to 369.9 pmol/L. There was no significant correlation between HE4 levels and neonates' gender. In the adult population, the research in this field is not conclusive. Lamy et al. confirmed the results obtained in this study, but Hertlein et al. suggested that the serum HE4 levels differed between genders [22, 23].

The HE4 concentrations during the delivery were also studied. The mean HE4 level in the mothers was 49.7 pmol/L with 95% confidence intervals for the median of 45.6 pmol/L to 57.9 pmol/L. Similar results were presented by other authors [24, 25].

The importance of this study lies in the fact that the HE4 levels were measured for the first time in the umbilical cord blood samples. The significantly higher umbilical HE4 levels in the entire study group were observed in comparison with the mothers' blood levels. It can be assumed that the majority of HE4 is of fetal origin in the cord blood, as this marker is not produced by the placenta [26].

An important finding was the increase in the neonatal HE4 levels determined 24 hours after the delivery. Under physiological conditions, the highest expression of the WFDC2 gene occurs in the lung tissue and endocrine glands [12, 13, 26]. During the delivery, there occur hormonal changes which probably stimulate the expression of this gene in the endocrine glands, which translates into the elevation of the HE4 concentrations in the umbilical cord blood. The first breath and further respiration of the child stimulate the gene expression in the lung tissue, resulting in the higher HE4 levels in the neonate venous blood.

The previous research demonstrates that some oncofetal antigens (HE4, alpha-fetoprotein (AFP), and carcinoembryonic antigen (CEA)) exhibit the expected pattern of expression with high concentrations at birth, followed by rapid decreases to significantly lower concentrations. This pattern is likely explained with the fact that many of the analytes are expressed during the fetal development and/or play an important role in the early postnatal development [20, 27]. It is also possible that these analytes are found in higher concentrations in the serum of neonates due to the immature hepatic function. In other words, if the liver processes these proteins, the high serum concentrations observed here may be the result of the inefficiency in immature neonatal livers [20].

Perhaps, HE4 plays an important role in the physiological fetal growth conditions, although it is recognized as a cancer biomarker. It may be similar to the alpha-fetoprotein, whose concentration is determined in the physiological pregnancy. However, the most characteristic increase is observed in malignant tumors, e.g., liver, testes, and ovaries [28].

In this study, higher levels of HE4 in the neonates with respiratory disorder were observed. It is well known that an inflammatory process may play an important role in the respiratory disorders (including TTN) [29]. Thanks to the previously published articles, it is acknowledged that the WDFC protein (to which HE4 belongs) is overexpressed in the inflammatory process. Therefore, it should be assumed that the higher HE4 concentrations observed in the newborn babies with TTN may be the result of the inflammatory process within the lungs. The obtained observations were confirmed with the ROC analysis results, which demonstrated not very high sensitivity and specificity for the early diagnosis of pulmonary dysfunction in the neonates due to the determination of the serum HE4 levels. We believe that serum HE4 levels could be considered as a candidate for early biochemical marker of respiratory insufficiency in newborns.

## 5. Conclusion

The obtained results may lead to a conclusion that the serum HE4 could be considered as a candidate biomarker for the early diagnosis of pulmonary dysfunction in newborns. This study is the first one to focus on the HE4 concentration analysis in the neonatal population; thus, its results should be confirmed with further studies in this field.

## Figures and Tables

**Table 1:** Demographic and perinatal characteristics of the investigated neonate groups. Results presented as a median and [95% confidence interval for median] or percentile.

Investigated group	Neonates with TTN (*n* = 23)	Controls (*n* = 28)	*p* value
Mothers' age (years)	29 [26-33]	30 [26-31]	0.34
Gestational age (weeks)	38 [37-39]	39 [38-40]	0.62
Gender: female (no.; %)	9; 39.1%	12; 42.9%	0.21^∗^
Gender: male (no.; %)	14; 60.9%	16; 57.1%	0.38^∗^
Birth weight (g)	3240 [3010-3510]	3145 [3040-3320]	0.46
Apgar score (no.; %)			
(i) 0-3 points	0	0	
(ii) 4-7 points	5; 21.7%	5; 17.9%	
(iii) 8-10 points	18; 78.3%	23; 82.1%	0.33^∗^

^∗^The *p* value from the Mann-Whitney *U* test or chi-squared test.

**Table 2:** Serum levels of human epididymis protein 4 (HE4), C-reactive protein (CRP), and procalcitonin (PCT) in the mothers' blood samples, umbilical cord blood samples, and neonatal blood samples collected 24 hours after the delivery. Results presented as a median and [95% confidence interval for median].

Investigated group	TTN (*n* = 23)	Controls (*n* = 28)	*p* value
Mothers' HE4 levels (pmol/L)	52.0 [42.8–74.8]	48.7 [43.8–63.1]	0.65
Umbilical HE4 levels (pmol/L)	235.8 [193.9–271.1]	204.5 [186.2–238.5]	0.14
Neonatal HE4 levels (pmol/L)	383.1 [312.8–511.7]	305.4 [284.3–370.1]	0.003
*p* value	<0.0001^∗^	<0.001^∗^	
Mothers' CRP levels (mg/L)	4.6 [3.2–6.3]	5.2 [3.4–6.0]	0.52
Mothers' PCT levels (ng/mL)	0.4 [0.1–0.4]	0.3 [0.1–0.3]	0.62
Neonatal CRP levels (mg/L)	1.8 [0.9–3.1]	1.3 [0.7–2.1]	0.11
Neonatal PCT levels (ng/mL)	0.6 [0.4–1.2]	0.7 [0.5–1.4]	0.45

^∗^The *p* value from the Mann-Whitney *U* test and Kruskal-Wallis test.

**Table 3:** Serum HE4 levels from the umbilical cord blood samples and neonatal blood samples collected 24 hours after the delivery, depending on the neonates' gender. Results presented as a median and [95% confidence interval for median]; the *p* value from the Mann-Whitney *U* test.

Investigated group	Female neonates (*n* = 21)	Male neonates (*n* = 30)	*p* value
Neonatal HE4 levels (pmol/L)	350.8 [285.3-450.4]	322.0 [250.1-406.8]	0.48
Umbilical HE4 levels (pmol/L)	257.9 [221.2-286.3]	227.2 [195.7-271.3]	0.33

## Data Availability

The data used to support the findings of this study are available from the corresponding author upon request.
